# Effects of Maternal LPS Exposure during Pregnancy on Metabolic Phenotypes in Female Offspring

**DOI:** 10.1371/journal.pone.0114780

**Published:** 2014-12-05

**Authors:** Xiao-Jing Liu, Bi-Wei Wang, Mei Zhao, Cheng Zhang, Yuan-Hua Chen, Chun-Qiu Hu, Hui Zhao, Hua Wang, Xi Chen, Fang-Biao Tao, De-Xiang Xu

**Affiliations:** 1 Department of Toxicology, Anhui Medical University, Hefei, China; 2 Anhui Provincial Key Laboratory of Population Health & Aristogenics, Anhui Medical University, Hefei, China; 3 First Affiliated Hospital, Anhui Medical University, Hefei, China; 4 Second Affiliated Hospital, Anhui Medical University, Hefei, China; University of Modena & Reggio Emilia, Italy

## Abstract

It is increasingly recognized that intra-uterine growth restriction (IUGR) is associated with an increased risk of metabolic disorders in late life. Previous studies showed that mice exposed to LPS in late gestation induced fetal IUGR. The present study investigated the effects of maternal LPS exposure during pregnancy on metabolic phenotypes in female adult offspring. Pregnant mice were intraperitoneally injected with LPS (50 µg/kg) daily from gestational day (GD)15 to GD17. After lactation, female pups were fed with standard-chow diets (SD) or high-fat diets (HFD). Glucose tolerance test (GTT) and insulin tolerance test (ITT) were assessed 8 and 12 weeks after diet intervention. Hepatic triglyceride content was examined 12 weeks after diet intervention. As expected, maternal LPS exposure during pregnancy resulted in fetal IUGR. Although there was an increasing trend on fat mass in female offspring whose dams were exposed to LPS during pregnancy, maternal LPS exposure during pregnancy did not elevate the levels of fasting blood glucose and serum insulin and hepatic triglyceride content in female adult offspring. Moreover, maternal LPS exposure during pregnancy did not alter insulin sensitivity in adipose tissue and liver in female adult offspring. Further analysis showed that maternal LPS exposure during pregnancy did not exacerbate HFD-induced glucose tolerance and insulin resistance in female adult offspring. In addition, maternal LPS exposure during pregnancy did not aggravate HFD-induced elevation of hepatic triglyceride content in female adult offspring. In conclusion, LPS-induced IUGR does not alter metabolic phenotypes in adulthood.

## Introduction

Lipopolysaccharide (LPS) is a toxic component of cell walls in gram-negative bacteria and is widely present in the digestive tracts of humans and animals [1]. Humans are constantly exposed to low levels of LPS through infection. Gastrointestinal distress and alcohol drinking often increase permeability of LPS from gastrointestinal tract into blood [2]. Increasing evidence demonstrated that maternal LPS exposure at different gestational stages was associated with adverse pregnant outcomes in rodent animals. According to an earlier report, pregnant mice exposed to LPS at early gestational stage caused embryonic resorption [3]. Recently, we found that pregnant mice exposed to LPS at middle gestational stage caused neural tube defects [4], [5]. Several studies showed that pregnant mice exposed to LPS at late gestational stage induced preterm delivery and fetal demise [6]–[11]. We and others demonstrated that pregnant mice exposed to LPS at late gestational stage resulted in fetal intra-uterine growth restriction (IUGR) [12]–[16].

It is increasingly recognized that fetal IUGR is associated with an increased risk of metabolic disorders like insulin resistance and diabetes mellitus, obesity, hypertension and cardiovascular diseases in late life [17]–[20]. Based on epidemiological data, Barker and coworkers described low weight at birth as highly correlated with increased risk for the development of cardiovascular diseases [21]. Further studies demonstrated that prenatal exposure to famine during late gestation, which resulted in IUGR, was linked to glucose tolerance in adults [22], [23]. The association between fetal IUGR and metabolic disorders in late life has also been demonstrated in animal experiments [24]. According to an earlier report, uteroplacental insufficiency and subsequent IUGR leads to altered hepatic fatty acid metabolism in adulthood [25]. A recent study showed that maternal protein restriction during pregnancy, which resulted in fetal IUGR followed by a rapid catch-up growth, obviously altered gene expression program in adipose tissue, leading to obesity in adult mice [26]. Nevertheless, it needs to be determined whether maternal LPS exposure during pregnancy, which also results in fetal IUGR, influences metabolic phenotypes in adult offspring.

In the present study, we hypothesize that LPS-induced IUGR alters metabolic phenotypes in late life, and increases the susceptibility of high-fat diet (HFD)-induced obesity, insulin resistance and fatty liver in adulthood. Thus, the aim of the current study was to investigate the effects of maternal LPS exposure during pregnancy on metabolic phenotypes in female adult offspring. In addition, we were also to explore whether maternal LPS exposure during pregnancy exacerbates HFD-induced metabolic disorders in female adult offspring.

## Materials and Methods

### Chemicals and reagents

Lipopolysaccharides (LPS) were purchased from Sigma Chemical Co. (St. Louis, MO). Anti-Akt and phospho-Akt-Ser473 were from Cell Signaling Technology (Beverley, MA). Insulin ELISA kit was from EMD Millipore Corporation (Millipore, MA). TNF-α ELISA kit was from R & D Systems (Minneapolis, MN). Horseradish peroxidase-conjugated goat anti-rabbit IgG was from Santa Cruz Biotechnology, Inc (Santa Cruz, CA). Chemiluminescence (ECL) detection kit was from Pierce Biotechnology (Rockford, IL) and polyvinylidene fluoride (PVDF) membrane was from Milipore Corporation (Belford, MA). All the other reagents were from Sigma or as indicated in the specified methods.

### Animals and treatments

The ICR mice (8–10 week-old; male mice: 28–30 g; female mice: 24–26 g) were purchased from Beijing Vital River whose foundation colonies were all introduced from Charles River Laboratories, Inc (Wilmington, MA, USA). The animals were allowed free access to food and water at all times and were maintained on a 12 h light/dark cycle in a controlled temperature (20–25°C) and humidity (50 ± 5%) environment for a period of 1 week before use. For mating purposes, four females were housed overnight with two males starting at 9:00 p.m. Females were checked by 7:00 a.m. the next morning, and the presence of a vaginal plug was designated as gestational day (GD) 0. Twenty pregnant mice were divided randomly into two groups. In the LPS group, pregnant mice were intraperitoneally (i.p.) injected with LPS(50 µg/kg) daily from GD15 to GD17. The 50 µg/kg dose of LPS was chosen since preliminary experiment showed that this LPS dosing regimen resulted in fetal IUGR but not preterm delivery and fetal demise. In the control group, pregnant mice were i.p. injected with NS daily from GD15 to GD17. After birth, litter sizes were standardized to 8 pups per litter. Throughout pregnancy and lactation, maternal mice were fed with standard-chow diets (SD, AIN-93, 3.41 kcal/g, 16.7% calories from fat). At weaning [postnatal week (PNW)3], female offspring were randomly assigned into four groups (each group comprised 20 animals) as follows: the SD group, female offspring from the control group were fed with standard-chow diets; the LPS group, female offspring from the LPS group were fed with standard-chow diets; the HFD group, female offspring from the control group were fed with high fat diets (4.73 kcal/g, 45% calories from fat) [27]; the LPS+HFD group, female offspring from the LPS group were fed with high fat diets. All diets were purchased from TROPHIC Animal Feed High-tech Co. Ltd (Nantong, Jiangsu, China). Female offspring were inspected daily for food intake and weighted weekly. Four weeks after diet intervention, fasting blood glucose was measured every two week. Intraperitoneal glucose tolerance test (IPGTT) and insulin tolerance test (ITT) were assessed 8 and 12 weeks after diet intervention. Fourteen weeks after diet intervention, all animals were anesthetized with phenobarbital sodium (50 mg/kg) and sacrificed. Blood serum was collected for biochemical parameters. Liver and visceral white adipose tissue were collected and either frozen immediately in liquid nitrogen for immunoblot and hepatic triglyceride measurement, or fixed in 4% paraformaldehyde for histology. This study was approved by the Association of Laboratory Animal Sciences and the Center for Laboratory Animal Sciences at Anhui Medical University (Permit Number: 13-0012). All procedures on animals followed the guidelines for humane treatment set by the Association of Laboratory Animal Sciences and the Center for Laboratory Animal Sciences at Anhui Medical University.

### IPGTT and ITT

IPGTT was performed after overnight fasting. For IPGTT, mice were i.p. injected with a single dose of glucose (2.0 g/kg) and blood glucose was measured using a glucometer (Roche Accu-Chek Inform) at different time points after glucose injection. ITT was performed after 4 h fasting. For ITT, mice were i.p. injected with a single dose of insulin (0.75 U/kg) and blood glucose was measured using a glucometer (Roche Accu-Chek Inform) at different time points after insulin injection.

### Immunoblots

To investigate insulin signaling in liver and adipose tissues, mice were fasted for 8 h and i.p. injected with insulin (1.0 U/kg). Mice were sacrificed 5 min after insulin injection. Liver and adipose tissues were quickly dissected and snap frozen in liquid nitrogen. Total lysate from liver or adipose tissues was prepared by homogenizing 50 mg liver or 200 mg adipose tissues in 300 µL ice-cold lysis buffer (50 mM Tris-HCl, pH 7.4, 150 mM NaCl, 1 mM EDTA, 1% Triton X-100, 1% sodium deoxycholate, 0.1% sodium dodecyl sulphate, 1 mM PMSF) supplemented with 1% cocktail of protease inhibitors (P8340, Sigma) and centrifuged at 14, 000 × g for 10 min at 4°C. The supernatant was collected and protein concentrations were determined with the BCA protein assay reagents. For immunoblot, same amount of protein (40 µg per lane) was separated by SDS-polyacrylamide gel electrophoresis (SDS-PAGE) and transferred onto a polyvinylidene fluoride membrane. The membranes were incubated for 2 h with either anti-Akt or phospho-Akt-Ser473 antibody. After washes in DPBS containing 0.05% Tween-20 four times for 10 min each, the membranes were incubated with goat anti-rabbit IgG antibody for 2 h. The membranes were washed for four times in DPBS containing 0.05% Tween-20 for 10 min each, followed by signal development using an ECL detection kit.

### Biochemical parameters

Triglyceride, total cholesterol, very low density lipoprotein (VLDL) cholesterol, alanine transaminase (ALT) and C-reactive protein were determined by routine laboratory methods using an auto-analyzer (Roche).

### Histology

Liver sections were stained with hematoxylin and eosin (H&E) to evaluate hepatic lipid accumulation. Adipose tissue sections were stained with H&E to assess the size and number of adipocytes in adipose tissue, respectively.

### Measurement for hepatic triglyceride

For hepatic triglyceride, liver samples were homogenized in ice-cold 2×PBS. Triglyceride was extracted with methanol/chloroform (1:2), dried, and resuspended in 5% fat-free bovine serum albumin. Hepatic triglyceride was measured using a commercially available kit according to manufacturer's protocol. Hepatic triglyceride content was expressed as µmol/g liver.

### Statistical analysis

Normally distributed data were expressed as means ± SEM. ANOVA and the Student-Newmann-Keuls post hoc test were used to determine differences among different groups. Data that were not normally distributed were assessed for significance using non-parametric test techniques (Kruskal-Wallis test and Mann-Whitney U test). *P* <0.05 was considered statistically significant.

## Results

### Effects of maternal LPS exposure during pregnancy on body weight and food consumption in female offspring

**Figure 1:**
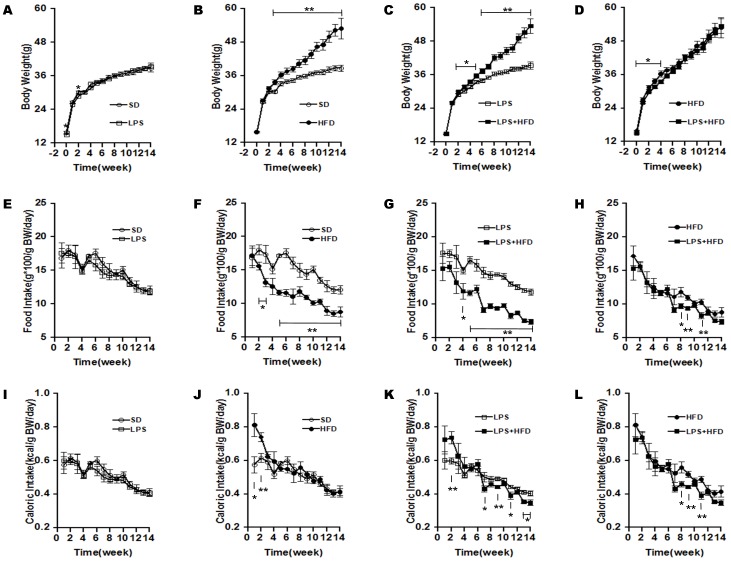
Effects of maternal LPS exposure during pregnancy on body weight growth and food intake in female offspring. Pregnant mice were i.p. injected with either LPS (50 µg/kg) or NS daily from GD15 to GD17. Throughout pregnancy and lactation, maternal mice were fed with standard-chow diets. After lactation, female offspring were assigned into four groups as follows: the SD group, from the NS group and fed with standard-chow diets; the LPS group, from the LPS group and fed with standard-chow diets; the HFD group, from the NS group and fed with high-fat diets; the LPS+HFD group, from the LPS group and fed with high-fat diets. Female offspring were inspected daily for food intake and weighted weekly. (A-D) Body weight. (E-H) Food intake. (I–L) Energy intake. Data were expressed as means ± SEM (n = 20). *P<0.05, **P<0.01.

The effects of maternal LPS exposure during pregnancy on body weight growth of female pups were analyzed. As expected, birth weight of newborn pups was significantly decreased in LPS-exposed mice (data not shown). Of interest, the body weight of female pups at weaning remained significantly lower in LPS-exposed mice as compared with controls (14.90 ± 0.23 g in the LPS group vs 15.80 ± 0.25 g in the control group, *P*<0.01). As shown in Figure 1A, a catch-up growth in standard-chow diet (SD)-fed female pups was observed until three weeks after diet intervention. The effects of high-fat diets on body weight growth of female pups are shown in Figures 1B and 1C. As expected, HFD-fed female pups showed a significantly accelerated elevation of body weight. Of interest, the difference of body weight between the LPS+HFD group and the LPS group reached statistical significant two weeks after diet intervention, one week earlier than that between the HFD group and the SD group. The effects of maternal LPS exposure during pregnancy on food and energy intakes of female pups were then analyzed. Despite a short catch-up growth in the LPS group, no significant difference on food and energy intakes was observed between the LPS group and the SD group (Figure 1E and 1I). Finally, the effects of high-fat diets on food and energy intakes were analyzed. Although HFD-fed female pups consumed fewer feed (Figures 1F and 1G), there was no significant difference on caloric intake between the HFD group and the SD group (Figures 1J and 1K). Further analysis showed that female pups in the LPS+HFD group consumed fewer food and caloric intakes in late stage as compared with the HFD group (Figures 1H and 1L).

### Effects of maternal LPS exposure during pregnancy on fat mass in female offspring

**Figure 2:**
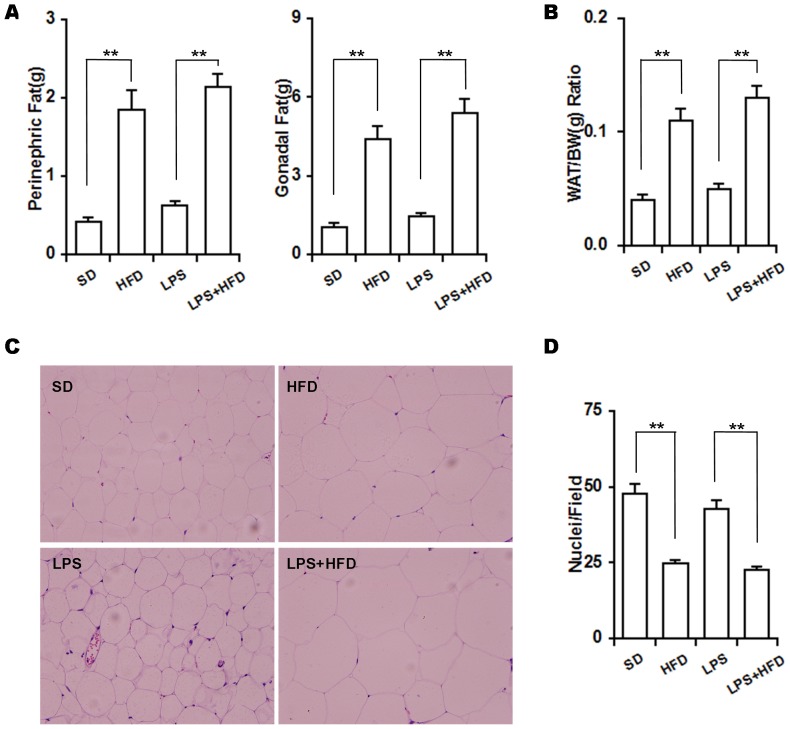
Effects of maternal LPS exposure during pregnancy on fat mass of female adult offspring. Pregnant mice were i.p. injected with either LPS (50 µg/kg) or NS daily from GD15 to GD17. Throughout pregnancy and lactation, maternal mice were fed with standard-chow diets. After lactation, female offspring were assigned into four groups as follows: the SD group, from the NS group and fed with standard-chow diets; the LPS group, from the LPS group and fed with standard-chow diets; the HFD group, from the NS group and fed with high-fat diets; the LPS+HFD group, from the LPS group and fed with high-fat diets. Fat mass was evaluated 14 weeks after diet intervention. (A) The weights of perinephric and gonadal fats. (B) White adipose tissue weight/body weight ratio. (C) Fatty sections were stained with H & E (Original magnification, ×200). (D) The number of nuclei per field was counted. WAT, white adipose tissue; BW, body weight. Data were expressed as means ± SEM (n = 20). *P<0.05, **P<0.01.

The effects of high-fat diets on fat mass were evaluated. As expected, perinephric and gonadal fats mass was significantly increased in the HFD group as compared with the SD group (Figure 2A). Accordingly, visceral white adipose mass/body weight ratio was elevated in the HFD group (Figures 2B). Histopathology showed that the size of adipocytes was markedly increased in the HFD group as compared with the SD group (Figure 2C). By contrast, the number of nuclei per field was significantly decreased in the HFD group (Figure 2D). The effects of maternal LPS exposure during pregnancy on fat mass in adipose tissue of female offspring were then analyzed. As shown in Figure 2A, there was an increasing trend on perinephric fat mass in the LPS group as compared with the SD group. Correspondingly, there was an increasing trend on fat index in female offspring whose dams were exposed to LPS during pregnancy (Figure 2B). Of interest, maternal LPS exposure during pregnancy had little effect on the size of adipocytes and the number of nuclei per field in female offspring (Figures 2C and 2D). Finally, we test whether maternal LPS exposure during pregnancy aggravates HFD-induced obesity in female offspring. As shown in Figures 2A and 2B, no statistical difference on fat mass was observed between the LPS+HFD group and the HFD group. Moreover, there was no significant difference in the size of adipocytes and the number of nuclei per field between the HFD group and LPS+HFD group (Figures 2C and 2D).

### Effects of maternal LPS exposure during pregnancy on hepatic and circulating lipids in female offspring

**Figure 3:**
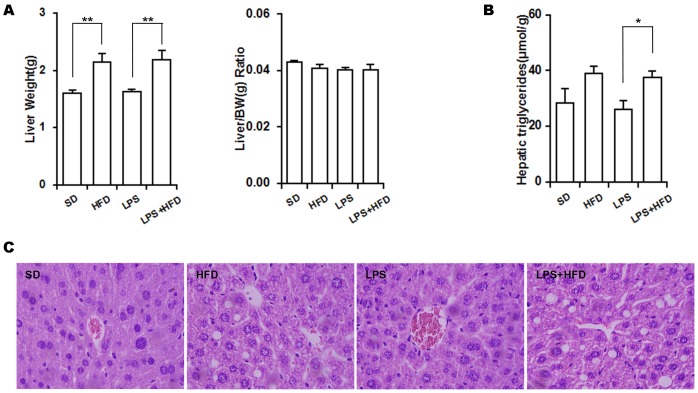
Effects of maternal LPS exposure during pregnancy on hepatic lipid accumulation and hepatic triglyceride content in female adult offspring. Pregnant mice were i.p. injected with either LPS (50 µg/kg) or NS daily from GD15 to GD17. Throughout pregnancy and lactation, maternal mice were fed with standard-chow diets. After lactation, female offspring were assigned into four groups as follows: the SD group, from the NS group and fed with standard-chow diets; the LPS group, from the LPS group and fed with standard-chow diets; the HFD group, from the NS group and fed with high-fat diets; the LPS+HFD group, from the LPS group and fed with high-fat diets. Hepatic triglyceride content and hepatic lipid accumulation were evaluated 14 weeks after diet intervention. (A) Liver weight (left) and liver weight/body weight ratio (right). (B) Hepatic triglyceride content. (C) Liver sections were stained with H & E (Original magnification, ×200). Data were expressed as means ± SEM (n = 20). *P<0.05, **P<0.01.

As expected, the absolute liver weight was significantly increased in the HFD group, whereas no significant difference on the relative liver weight was observed between HFD-fed and SD-fed female offspring (Figure 3A). The effects of maternal LPS exposure during pregnancy on liver weight of female offspring were analyzed. As shown in Figure 3A, maternal LPS exposure during pregnancy had no effect on the absolute and relative liver weights in female offspring. Moreover, maternal LPS exposure during pregnancy did not aggravate HFD-induced elevation of the absolute liver weight in female offspring (Figure 3A). The effects of maternal LPS exposure during pregnancy on hepatic triglyceride content and hepatic lipid accumulation in female offspring were then analyzed. As expected, hepatic triglyceride content was significantly increased in the HFD group as compared with the SD group (Figure 3B). Histopathology showed that hepatic lipid accumulation was observed in HFD-fed female offspring but not in SD-fed female offspring (Figure 3C). Interestingly, maternal LPS exposure during pregnancy had little effect on hepatic triglyceride content and hepatic lipid accumulation in female offspring (Figures 3B and 3C). Moreover, maternal LPS exposure during pregnancy did not aggravate HFD-induced elevation of hepatic triglyceride content in female offspring (Figure 3B). Correspondingly, maternal LPS exposure during pregnancy did not aggravate HFD-induced hepatic lipid accumulation in female offspring (Figure 3C). The effects of maternal LPS exposure during pregnancy on serum lipids are shown in Table 1. As expected, no significant differences were observed in serum triglyceride and VLDL cholesterol levels among different groups. Of interest, serum total cholesterol was significantly elevated in the HFD group as compared with the SD group, whereas maternal LPS exposure during pregnancy had no effect on serum total cholesterol in female offspring. Moreover, maternal LPS exposure during pregnancy did not aggravate HFD-induced elevation of serum total cholesterol in female offspring (Table 1).

**Table 1:** Serum biochemical parameters in female offspring.

Serum parameters	SD	LPS	HFD	LPS+HFD
ALT (U/l)	23.5±1.91	18.5±0.56	36.83±7.34	41.67±6.23^#^
Triglyceride (mM)	1.73±0.34	2.15±0.23	1.87±0.13	1.94±0.14
Total cholesterol (mM)	2.46±0.32	2.70±0.22	3.96±0.2**	4.47±0.24^##^
VLDL cholesterol (mM)	0.64±0.13	0.79±0.08	0.70±0.05	0.72±0.05
Glucose (mM)	4.42±0.28	4.83±0.23	6.11±0.36**	6.5±0.62^##^
Insulin (ng/ml)	0.66±0.08	0.79±0.12	0.95±0.09	1.16±0.15
C-reactive protein (mg/l)	0.50±0.05	0.50±0.07	0.51±0.07	0.44±0.03
TNF-α (pg/ml)	3.32±0.08	3.54±0.13	3.35±0.09	2.92±0.06

Glucose, Insulin, TNF-α, n = 20/group. Other parameters, n = 6/group. All results are expressed as mean±SEM. **P<0.01, versus Control. ^#^ *P*<0.05, ^##^ *P*<0.01, versus LPS.

### Effects of maternal LPS exposure during pregnancy on fasting blood glucose and serum insulin in female offspring

**Figure 4:**
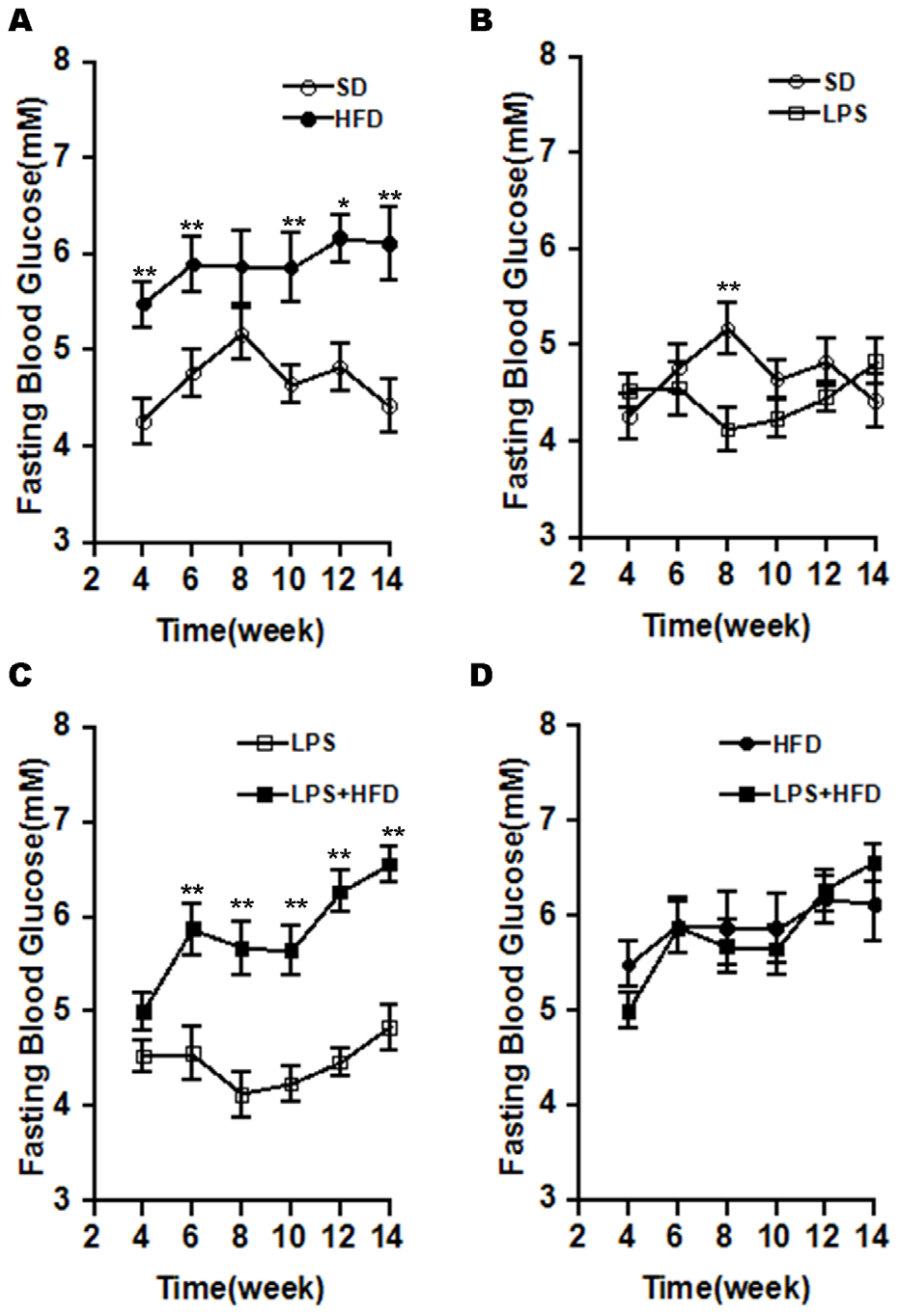
Effects of maternal LPS exposure during pregnancy on fasting blood glucose in female adult offspring. Pregnant mice were i.p. injected with either LPS (50 µg/kg) or NS daily from GD15 to GD17. Throughout pregnancy and lactation, maternal mice were fed with standard-chow diets. After lactation, female offspring were assigned into four groups as follows: the SD group, from the NS group and fed with standard-chow diets; the LPS group, from the LPS group and fed with standard-chow diets; the HFD group, from the NS group and fed with high-fat diets; the LPS+HFD group, from the LPS group and fed with high-fat diets. Fasting blood glucose was measured every two week. Data were expressed as means ± SEM (n = 20). *P<0.05, **P<0.01.

Fasting blood glucose was measured at different time points after diet intervention. As expected, fasting blood glucose level was significantly higher at all time points in the HFD group than in the SD group (Figure 4A). The effects of maternal LPS exposure during pregnancy on fasting blood glucose level of female offspring were analyzed. Although fasting blood glucose level at the eighth week after diet intervention was significantly decreased in the LPS group, no significant difference on fasting blood glucose level at other time points was observed between the LPS group and the SD group (Figure 4B). Moreover, maternal LPS exposure during pregnancy did not aggravate HFD-induced elevation of fasting blood glucose level in female offspring (Figure 4D). The effects of maternal LPS exposure during pregnancy on fasting serum insulin of female offspring were then analyzed 14 weeks after diet intervention. As expected, there were markedly upward trend on the level of fasting serum insulin in the HFD group (Table 1). Of interest, maternal LPS exposure during pregnancy had no effect on the level of fasting serum insulin. Moreover, maternal LPS exposure during pregnancy did not aggravate HFD-induced elevation of fasting serum insulin in female offspring (Table 1).

### Effects of maternal LPS exposure during pregnancy on glucose tolerance in female offspring

**Figure 5:**
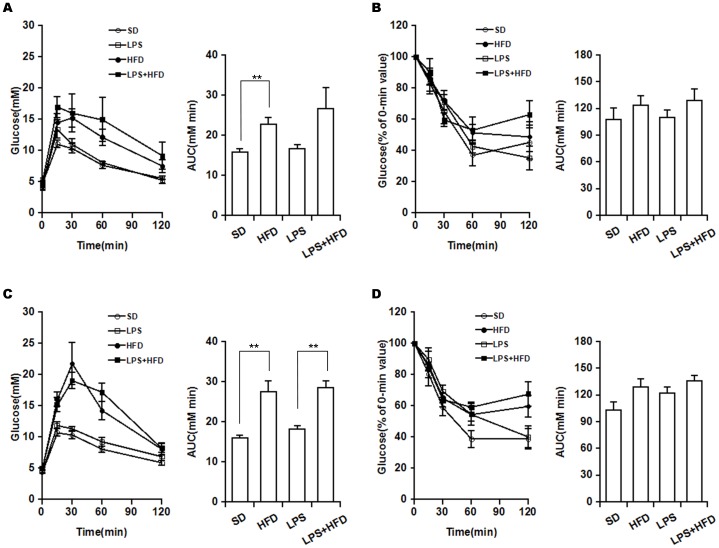
Effects of maternal LPS exposure during pregnancy on glucose and insulin tolerances in female adult offspring. Pregnant mice were i.p. injected with either LPS (50 µg/kg) or NS daily from GD15 to GD17. Throughout pregnancy and lactation, maternal mice were fed with standard-chow diets. After lactation, female offspring were assigned into four groups as follows: the SD group, from the NS group and fed with standard-chow diets; the LPS group, from the LPS group and fed with standard-chow diets; the HFD group, from the NS group and fed with high-fat diets; the LPS+HFD group, from the LPS group and fed with high-fat diets. Five mice in each group were chosen randomly for either IPGTT or ITT at 8 and 12 weeks after diet intervention. (A) IPGTT results at 8 weeks after diet intervention. (B) ITT results at 8 weeks after diet intervention. (C) IPGTT results at 12 weeks after diet intervention. (D) ITT results at 12 weeks after diet intervention. Data were expressed as means ± SEM (n = 5). *P<0.05, **P<0.01.

The effects of maternal LPS exposure during pregnancy on glucose tolerance in female offspring at 8 weeks after diet intervention were analyzed using IPGTT. As shown in Figure 5A, blood glucose levels were significantly increased among all groups in response to glucose injection. Compared with the control mice, HFD-fed female offspring presented a significant elevation of the glucose area under the curve during the IPGTT (Figure 5A). Interestingly, maternal LPS exposure during pregnancy did not exert a significant influence on glucose tolerance in either SD or HFD-fed female offspring (Figure 5A). The effects of maternal LPS exposure during pregnancy on glucose tolerance of female offspring at 12 weeks after diet intervention are presented in Figure 5C. Consistent with the results of IPGTT at the eighth week, HFD-fed female offspring showed an obvious elevation of the glucose area under the curve during the IPGTT, but maternal LPS exposure during pregnancy did not produce a significant influence on glucose tolerance in either SD or HFD-fed female offspring.

### Effects of maternal LPS exposure during pregnancy on insulin tolerance in female offspring

The effects of maternal LPS exposure during pregnancy on insulin tolerance of female offspring at 8 weeks after diet intervention were analyzed using ITT. As shown in Figure 5B, blood glucose levels were significantly decreased among all groups in response to insulin injection. Compared with SD-fed mice, HFD-fed female offspring presented a tendency to an increase in the glucose area under the curve during the ITT (Figure 5B). However, this change did not reach statistical significance. Moreover, maternal LPS exposure during pregnancy did not exert a significant influence on insulin sensitivity in either SD-fed or HFD-fed female offspring (Figure 5B). The effects of maternal LPS exposure during pregnancy on insulin tolerance of female offspring at 12 weeks after diet intervention are presented in Figure 5D. Consistent with the results of ITT at the eighth week, HFD-fed female offspring showed a tendency to an increase in the glucose area under the curve during the ITT. Although LPS-exposed female offspring showed a tendency to an increase in the glucose area under the curve, maternal LPS exposure during pregnancy did not aggravate HFD-induced impairment for insulin sensitivity in female offspring.

### Effects of maternal LPS exposure during pregnancy on Insulin signal transduction in adipose tissue and liver of female offspring

**Figure 6:**
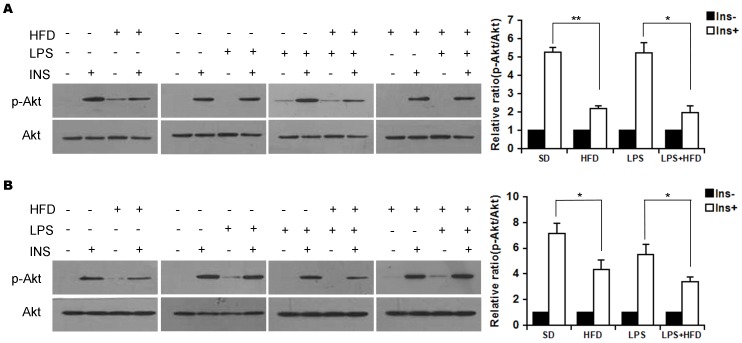
Effects of maternal LPS exposure during pregnancy on Akt phosphorylation in adipose tissue and liver of female adult offspring. Pregnant mice were i.p. injected with either LPS (50 µg/kg) or NS daily from GD15 to GD17. Throughout pregnancy and lactation, maternal mice were fed with standard-chow diets. After lactation, female offspring were assigned into four groups as follows: the SD group, from the NS group and fed with standard-chow diets; the LPS group, from the LPS group and fed with standard-chow diets; the HFD group, from the NS group and fed with high-fat diets; the LPS+HFD group, from the LPS group and fed with high-fat diets. Akt and p-Akt in adipose tissue and liver were measured using immunoblots 14 weeks after diet intervention. (A) p-Akt/Akt in adipose tissue. (B) Hepatic p-Akt/Akt. All experiments were replicated for four times. Data were expressed as means ± SEM. *P<0.05, **P<0.01.

To investigate the effects of maternal LPS exposure during pregnancy on insulin signal transduction in female offspring, p-Akt and Akt were measured in adipose tissue and liver. The effects of maternal LPS exposure during pregnancy on p-Akt and Akt in adipose tissue are shown in Figure 6A. Although the levels of Akt protein in adipose tissue did not differ among different groups, the level of p-Akt in adipose tissue was significantly increased in response to insulin challenge. As expected, the level of p-Akt in adipose tissue was significantly decreased in HFD-fed mice. Of interest, maternal LPS exposure during pregnancy had no effect on the level of p-Akt in adipose tissue of female offspring. Moreover, maternal LPS exposure during pregnancy did not aggravate HFD-induced reduction of p-Akt level in adipose tissue of female offspring. The effects of maternal LPS exposure during pregnancy on hepatic p-Akt and Akt are presented in Figure 6B. Although the levels of hepatic Akt protein did not differ among different groups, the level of hepatic p-Akt was significantly increased in response to insulin challenge. As expected, the level of hepatic p-Akt was significantly decreased in HFD-fed mice. Of interest, maternal LPS exposure during pregnancy had no effect on the level of hepatic p-Akt in female offspring. Moreover, maternal LPS exposure during pregnancy did not aggravate HFD-induced reduction of hepatic p-Akt level in female offspring.

## Discussion

In the present study, we showed that birth weight was significantly decreased in newborn pups whose dams were exposed to LPS during pregnancy. These results are in agreement with others, in which maternal LPS exposure at late gestational stage resulted in fetal IUGR in rodent animals [12]–[16], [28] Several reports demonstrated that pups from dams that were restricted to food during pregnancy had lower birth weights than controls, followed by a rapid catch-up growth as early as in the suckling period [29], [30]. Of interest, the present study showed that body weight of female pups at weaning remained significantly lower in LPS-exposed mice as compared with controls. Although a much slow catch-up growth in standard-chow diet-fed female pups was observed until three weeks after diet intervention (PNW6), no significant elevation of body weight in adulthood was observed in LPS-exposed mice as compared with controls. These results suggest that maternal LPS exposure at late gestational stage resulted in fetal IUGR followed by a much slow catch-up growth.

According to an earlier epidemiological investigation, an impaired insulin sensitivity was observed in small for gestational age (SGA) children, which may contribute to enhanced risk of type II diabetes in adulthood, especially in SGA children with catch-up growth and a high body mass index (BMI) [31]. Numerous animal experiments demonstrated that IUGR rats predisposed to develop obesity and insulin resistance early in life and overt diabetes in adulthood that was characterized by fasting hyperglycemia and hyperinsulinemia [32], [33]. The present study investigated the effects of maternal LPS exposure during pregnancy on the levels of fasting blood glucose and serum insulin, glucose and insulin tolerances, and insulin signal transduction in adipose tissue and liver in female adult offspring. Unexpectedly, although there was an increasing trend on fat mass in female adult offspring from the LPS group as compared with the SD group, maternal LPS exposure during pregnancy did not elevate the levels of fasting blood glucose and serum insulin in female adult offspring. Moreover, maternal LPS exposure during pregnancy did not induce glucose tolerance, as determined by IPGTT, and insulin tolerance, as determined by ITT, in female adult offspring. In addition, maternal LPS exposure during pregnancy did not alter insulin signal transduction in adipose tissue and liver in female adult offspring. These results suggest that maternal LPS exposure during pregnancy does not impair insulin sensitivity in adipose tissue and liver in female adult offspring.

An earlier report showed that IUGR, caused by uteroplacental insufficiency, altered the expression of hepatic fatty acid-metabolizing enzymes in juvenile and adult rats [25]. A recent study demonstrated that prenatal hypoxia, which induced fetal IUGR, predisposed to develop hepatic lipid accumulation in adult rats [34]. To investigate whether maternal LPS exposure during pregnancy influences lipid metabolism in late life, the present study measured hepatic and circulating lipids in female adult offspring. Interestingly, maternal LPS exposure during pregnancy had little effect on hepatic triglyceride content in female adult offspring. Moreover, maternal LPS exposure during pregnancy did not induce hepatic lipid accumulation in female adult offspring. In addition, maternal LPS exposure during pregnancy did not elevate the level of serum triglyceride, total cholesterol and VLDL cholesterol in female adult offspring. These results suggest that maternal LPS exposure during pregnancy does not alter lipid metabolism in female adult offspring.

Recently, several studies demonstrated that IUGR markedly increased the susceptibility of rats to high-fat diet-induced metabolic syndromes including obesity, insulin resistance and fatty liver [35]–[37]. As maternal LPS exposure during pregnancy results in IUGR, the present study test whether maternal LPS exposure during pregnancy exacerbates HFD-induced obesity, glucose tolerance, insulin resistance and hepatic lipid accumulation in adulthood. Unexpectedly, maternal LPS exposure during pregnancy had little effect on HFD-induced obesity and elevation of circulating lipids in female adult offspring. Moreover, maternal LPS exposure during pregnancy did not aggravate HFD-induced increase in hepatic triglyceride content in female adult offspring. In addition, maternal LPS exposure during pregnancy did not exacerbate HFD-evoked glucose tolerance and insulin resistance in female adult offspring. These results suggest that LPS-induced IUGR does not increase the susceptibility of mice to high-fat diet-induced metabolic syndromes.

Epidemiological data have demonstrated that low birth weight (LBW) and SGA children, who subsequently show a rapid catch-up growth, have higher susceptibility for the development of obesity, type 2 diabetes and cardiovascular diseases later in life [38]–[43]. In rats, a catch-up growth immediately after food restriction is essential for the development of obesity and insulin resistance in adulthood [44]–[46]. According to a recent study, pups from both dams that were restricted to food and that were exposed to dexamethasone during pregnancy had similarly lower birth weight than controls, but only food restriction led to a rapid catch-up growth, which occurred as early as in the suckling period, and later development of glucose intolerance in adulthood [47]. Of interest, the present study showed that maternal LPS exposure during pregnancy resulted in fetal IUGR followed by a much slow catch-up growth in female pups, which occurred until three weeks after diet intervention (PNW6). Moreover, no significant elevation of body weight in adulthood was observed in LPS-exposed mice as compared with controls. In addition, maternal LPS exposure during pregnancy had no effect on food and energy intakes in female offspring. Actually, female pups in the LPS+HFD group consumed fewer food and caloric intakes in late stage as compared with the HFD group. These results partially explain why LPS-induced IUGR does not alter metabolic phenotypes in adulthood.

A recent study found that LPS exposure during pregnancy elevated corticosterone levels in maternal serum but not in adult offspring [48]. Moreover, maternal LPS exposure during pregnancy modified the immune response to a LPS challenge in adult offspring, which might contribute to LPS-induced autistic-like behavioral impairment [48]. The present study laid emphasis on the effects of maternal LPS exposure, which mimics infections by gram-negative bacteria during pregnancy, on metabolic phenotypes in female offspring. The present study has several major flaws: first, the present study only investigated whether LPS-induced IUGR influences metabolic phenotypes in early adulthood; second, the present study only investigated the effects of maternal LPS exposure during late pregnancy on metabolic phenotypes in offspring; third, the present study only investigated the effects of maternal LPS exposure during pregnancy on metabolic phenotypes in female adult offspring. Indeed, an earlier report showed that IUGR-associated metabolic disorders appeared to develop earlier in males than in females [49]. Another study demonstrated that there were early changes in the expression of peroxisome proliferator-activated receptor (PPAR)γ and leptin mRNA in visceral adipose tissue of IUGR males, which preceded the emergence of an obese phenotype [50]. Thus, additional study is necessary to further explore whether maternal LPS exposure during pregnancy alters metabolic phenotypes in male adult offspring. Thus, additional work is required to assess the effects of maternal LPS exposure at early and middle gestational stages on metabolic phenotypes in offspring. Moreover, further research is needed to explore the effects of maternal LPS exposure during pregnancy on metabolic phenotypes in male offspring. In addition, the effects of maternal LPS exposure during pregnancy on metabolic phenotypes in offspring need to be demonstrated in different animal models.

In summary, the present study investigated the effects of maternal LPS exposure during pregnancy on metabolic phenotypes in female adult offspring. Although maternal LPS exposure during pregnancy results in fetal IUGR, it does not impair insulin sensitivity in adipose tissue and liver of female adult offspring. Moreover, maternal LPS exposure during pregnancy does not influence hepatic triglyceride metabolism in female adult offspring. Importantly, maternal LPS exposure during pregnancy does not exacerbate HFD-induced obesity, glucose tolerance, insulin resistance and hepatic lipid accumulation in female adult offspring. Thus, LPS-induced IUGR does not alter metabolic phenotypes in female adult offspring.
